# Pediatric renal abscess: clinical analysis and literature review

**DOI:** 10.3389/fped.2025.1407437

**Published:** 2025-04-28

**Authors:** Jianxin Sun, Lina Shi, Lezhen Ye, Yanan Xu

**Affiliations:** ^1^ Department of Paediatrician, Women’s and Children’s Hospital of Ningbo University, Ningbo, China; ^2^ Department of Paediatrician, Yuyao Maternal and Child Health Centre, Yuyao, China; ^3^ Department of Scientific Research, Women’s and Children’s Hospital of Ningbo University, Ningbo, China

**Keywords:** renal, abscess, pediatric, management, review

## Abstract

**Background:**

Pediatric renal abscesses is a severe infectious disease with a long treatment period. Due to atypical symptoms, there is a risk of delayed diagnosis, missed diagnosis, and misdiagnosis. Inadequate or incomplete treatment can lead to prolonged hospital stays, even Irreversible kidney damage. This study aimed to analyze the clinical characteristics of pediatric renal abscesses, aiming for early diagnosis and timely, appropriate treatment.

**Methods:**

A retrospective analysis was conducted on clinical manifestations, laboratory tests, imaging studies, and treatment data of 12 pediatric renal abscess cases treated in the Nephrology Department of our hospital from October 2018 to March 2023.

**Results:**

Among the 12 cases, there were 3 males and 9 females, aged between 7 months to 12 years. All cases were from urban areas, with fever being the primary symptom (100%), accompanied in some by abdominal pain and urinary frequency/pain. Clinical symptoms were atypical, with 91% showing elevated white blood cell count(WBC), a significant rise in neutrophil percentage, C-reactive protein (CRP), and a marked increase in procalcitonin (100%). significant elevation of urinary white blood cells in 83.3% of cases. Both urine and blood cultures were negative. All 12 cases underwent abdominal CT or Magnetic Resonance Urography (MRU), showing abscesses, all less than 3 cm. Treatment included third-generation cephalosporins, with the addition of linezolid in cases where the initial treatment was ineffective. Hospital stays ranged from 10 to 21 days. Follow-up MRU showed the disappearance of abscesses.

**Conclusion:**

Clinical symptoms of pediatric renal abscesses are atypical. Children with fever, accompanied by abdominal pain, and significant elevation in white blood cells, CRP, and PCT should be considered for renal abscess, and abdominal CT or MRU is recommended for early diagnosis. Conservative anti-infection treatment can yield good results for abscesses smaller than 3 cm.

## Backgroud

Pediatric renal abscess is an acute localized purulent lesion of the renal parenchyma, which is very rare in children. Due to atypical symptoms, there is a risk of delayed diagnosis, missed diagnosis, and misdiagnosis. Inadequate or incomplete treatment can lead to prolonged hospital stays, even irreversible kidney damage,and endangering life. Compared with urinary tract infection, which is characterized by typical urinary tract irritation symptoms such as frequent urination, urgency, and painful urination, renal abscess has no specific clinical manifestations. Literature reports indicate that early diagnosis can be misidentified as respiratory infections, and even ultrasound may show localized masses mistaken for tumors. The infection pathways of renal abscess primarily involve ascending urinary tract infection or hematogenous spread, with Escherichia coli being the most common pathogen, followed by Staphylococcus in some cases. Regarding treatment, literature mainly recommends conservative management. This study aimed to analyze the clinical characteristics of pediatric renal abscesses, aiming for early diagnosis and timely, appropriate treatment.

## Materials and methods

### General information

This study is a retrospective analysis. The study was approved by the Hospital's Medical Ethics Committee (approval number: EC2023-011). Guardians of the children involved in the study were fully informed, consented to the research, and signed informed consent forms. Clinical data of 12 pediatric patients with renal abscesses treated in the Pediatric Nephrology Department of our hospital from October 2018 to March 2023 were collected.

### Methods

Clinical data of the pediatric patients were collected through the hospital's electronic medical record system. This included a range of information such as basic details (gender, age, living environment), clinical presentations, and laboratory tests (white blood cells, neutrophil percentage, C-reactive protein, calcitonin, erythrocyte sedimentation rate, serum creatinine, urine white blood cells, urine red blood cells, urine microprotein, urine culture, blood culture). Imaging studies like MRU, abdominal CT, urinary system ultrasound, and retrograde ureterography were also reviewed, along with treatment details. Patient outcomes were gathered through telephone follow-ups and outpatient medical record systems.

### Statistical analysis

Descriptive analysis was employed. Quantitative data such as age, white blood cells, neutrophil ratio, and serum creatinine were represented using the median (M) and range.Results are presented as mean ± standard deviation for continuous variables.

## Results

### Basic information

Among the 12 pediatric patients, there were 3 males and 9 females; ages ranged from 7 months to 12 years (median age 6 ± 3.86 years), including 2 cases under 1 year old (Table 1). Residential Environment: All patients came from urban areas with parents having a college degree or higher education.

**Table 1:** Clinical data and imaging, ultrasound, treatment, and outcomes of patients.

No.	Gender	Age (months)	Initial symptoms	MRU/CT findings	Urinary system ultrasound	MCU findings	hospital stay	Follow-up	Treatment
1	Female	7	Fever	Abscess in lower pole of right kidney	Bilateral renal cysts	Not done	21 days	3 weeks normal	Cefoperazone
2	Female	11	Fever	Abscess in upper pole of right kidney	No abnormalities	Not done	14 days	4 weeks normal	Ceftriaxone
3	Male	96	Fever, abdominal pain	Multiple patchy abnormal signals in both kidneys	No abnormalities	Not done	13 days	2 weeks normal	Cefoperazone
4	Male	144	Fever, abdominal pain	Multiple patchy abnormal signals on right side	Both kidneys enlarged	Not done	14 days	4 weeks normal	Cefoperazone
5	Female	52	Fever	Right kidney abscess	Right kidney low echo	No reflux	17 days	4 weeks normal	Cefoperazone, Linezolid
6	Female	63	Fever, abdominal pain	Infection with abscess in lower pole of right kidney	No abnormalities	Not done	10 days	3 weeks normal	Cefoperazone, Linezolid
7	Female	40	Fever, right renal mass	Right kidney abscess	Moderate echogenicity of right kidney	Not done	20 days	1 year: Reduced renal abscess	Cefoperazone, Linezolid
8	Female	120	Fever	Infection with abscess in right kidney	Upper pole of right kidney low echo	Not done	14 days	2 months normal	Cefoperazone, Linezolid
9	Male	84	Fever, urinary pain	Multiple patchy abnormal signals in both kidneys	Thickened bladder wall	Bilateral grade 3 reflux	10 days	4 weeks normal	Cefoperazone, Linezolid
10	Female	21	Fever, urinary frequency	Multiple enhancing lesions in left kidney	Enlarged left kidney	Not done	10 days	2 months normal	Cefotaxime, Cefoperazone
11	Female	105	Fever, abdominal pain, vomiting	Abscess in upper pole of right kidney	No abnormalities	No reflux	18 days	4 weeks normal	Cefotaxime
12	Female	115	Fever, abdominal pain	Abnormal signal in left kidney	Moderate echogenicity of left kidney	Not done	21 days	4 months: Reduced renal abscess	Cefoper

### Clinical symptoms

All patients primarily presented with fever (100%), predominantly high fever (body temperature ≥39°C). The duration of illness before admission was 1–8 days (median course 4 ± 0.11 days), with no history of preceding infections. Five cases were accompanied by abdominal pain, two cases exhibited urinary frequency or urinary pain, one case had a right renal mass, three cases were transferred from the gastroenterology department, and one case from the surgery department. Only one child presented with renal percussion pain (Table 1).

### Laboratory and imaging examinations

Renal ultrasound was performed on all patients upon admission, with only 4 cases (33.33%) showing renal abscesses. All 12 patients underwent abdominal CT or MRU, revealing abscesses (8 on the right side (66.67%), 2 bilateral (16.67%), and 2 on the left side (16.67%), all less than 3 cm in size). Three patients underwent Micturating Cystourethrography (MCU), of which 2 showed no reflux, and 1 exhibited bilateral grade 3 reflux (Table 1). Peripheral blood WBC ranged from (11.8 to 33) × 10^9^/L, neutrophil percentage (*N*) from 67 to 91%, CRP from 11 to 239 mg/L, and calcitonin from 0.49 to 15 ng/ml. Kidney function tests showed no abnormalities. Urinalysis indicated red blood cells (RBC) ranging from 4 to 26 per HP, white blood cells (WBC) from 0 to 528 per HP, and urinary red blood cells from 0 to 26 per HP. Both urine and blood cultures were negative (Table 2). Laboratory data showed that 91% of the children had elevated white blood cell counts, with a significant increase in the proportion of neutrophils. C-reactive protein (CRP) and erythrocyte sedimentation rate (ESR) were markedly elevated in 66% of the cases. Calcitonin levels were significantly increased in all cases (100%). Urinalysis revealed that 83.3% of the patients had a notable increase in urinary white blood cells. Hematuria was present in 50% of the cases, and the rate of positive urinary protein was 50% (Table 3).

**Table 2:** Laboratory data.

No.	WBC (×10^9^/L)	Neutrophils (%)	CRP (mg/L)	Calcitonin (ng/ml)	ESR (mm/h)	Creatinine (µmol/L)	Urine WBC (/HP)	Urine RBC (/HP)	Urine tetra protein	Urine Culture	Blood Culture
1	11.8	76	68	14.29	36	23	25	4	Negative	–	-
2	24.9	67	11	0.49	34	21	528	4	Positive	–	-
3	13	71	0.6	1.59	12	63	2.7	8	Negative	–	-
4	13.9	90	222	8.2	97	80	14	4.8	Positive	–	-
5	15.9	75	89	0.62	88	47	0	–	Positive	–	-
6	20.9	81	107	4.5	39	59	136	7	Negative	–	-
7	12	60	0.07	0.6	32	23	6	–	Negative	–	-
8	19.6	19	129	0.5	78	59	11	–	Negative	–	-
9	25.8	84	45	0.6	20	45	196	26	Negative	–	-
10	33	69	86	11	30	32	9	2	Positive	–	-
11	10	79	107	0.6	18	-	6	2	Positive	–	-
12	16	91	239	15	46	-	12	0	Positive	–	-

WBC, white blood cell; CRP, C-reactive protein; RBC, red blood cell; ESR, erythrocyte sedimentation rate.

**Table 3:** Laboratory data overview.

Laboratory parameter	Number of cases (*n* = 12)	Percentage (%)
Elevated WBC	11	91
Elevated neutrophils	7	58
Elevated CRP	10	83
Elevated calcitonin	12	100
Elevated ESR	10	83
Elevated urine WBC	10	83
Elevated urine RBC	6	50
Positive urine protein	6	50
Negative urine culture	0	0
Negative blood culture	0	0

Elevated WBC > 10 × 10^9^/L, Elevated CRP > 8 mg/L, Elevated calcitonin > 0.05 ng/ml, Elevated ESR > 20 mm/h, Elevated urine WBC > 5/HP, Elevated RBC > 3/HP.

### Treatment

**Figure 1:**
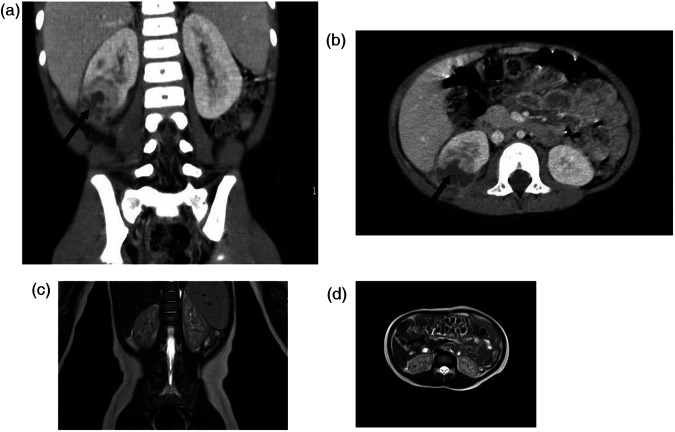
The changes of CT before treatment **(a,b)**, and MRU after about 1 month of treatment **(c,d)** in case five. **(a,b)** The abscesses in lower pole of the right kidney were measured as about 2.4 × 2.7 cm (arrow), **(c,d)** The abscesses was largely disappeared.

Upon admission, patients were treated with third-generation cephalosporin antibiotics. This treatment was effective in 7 cases, while 5 cases showed no response and were subsequently treated with linezolid. All patients received conservative treatment. The length of hospital stay ranged from 10 to 21 days. Within 2–10 days of treatment, the children's body temperature normalized. At 2 weeks to about 1 month post-discharge, a follow-up renal MRU in 8 cases showed the disappearance of renal abscesses (Figure 1). In 2 cases, abscesses completely resolved after continued medication for 2 months post-discharge. And the total duration of palliative antibiotic therapy was 14–60 days (median course 28 ± 7.30 days).In 1 case, the abscess size reduced after 4 months, and in 1 child, a small lesion was detected during a 1-year follow-up ultrasound (with normal urinalysis). One child with reflux underwent surgical treatment and was normal at the 1-year follow-up (Table 1).

## Discussion

Intra-abdominal abscesses are relatively rare in children, with renal abscesses being even rarer. A retrospective analysis study found that among approximately 200,000 pediatric emergency department visits, there were only 17 cases of renal abscesses. The incidence rate is about 0.007% (1). However, it is a severe infectious disease with a long treatment period and significant damage to renal parenchyma. Due to atypical symptoms, there is a risk of delayed diagnosis, missed diagnosis, and misdiagnosis. Inadequate or incomplete treatment can lead to prolonged hospital stays (2, 3). Literature review reveals no related guidelines; diagnosis and treatment are based on adult and small-sample data, with limited case information and no unified diagnostic and therapeutic strategies (4).

Children of all ages can be affected. An 11-year literature retrospective study found that the age of onset ranged from 1 month to 18 years, more common in girls (2, 4, 5), though some reports indicate a higher proportion in boys (6, 7). Our data show that the age of affected children in our group ranged from 7 months to 12 years (median age 6 ± 3.86 years), with a higher proportion in girls. This suggests that renal abscesses may not have a significant gender difference. Additionally, whether the incidence of renal abscesses is related to residential areas and living environments is unclear. Our group of children all came from urban areas, with parents having college-level education or higher, and living in good environmental conditions. This suggests that the occurrence of renal abscesses may not be related to living environments, but further accumulation of clinical data is needed for observation.

The clinical presentation of pediatric renal abscesses lacks specificity. Most cases present with fever as the main clinical manifestation, while some children experience back pain, abdominal pain, and abdominal masses (8, 9). Thus, patients are not initially admitted to nephrology or urology departments but are dispersed in other departments, such as initially being admitted to gastroenterology, general surgery, or other pediatric departments, diagnosed with “surgical diseases such as appendicitis, digestive system diseases, sepsis”, etc. (2, 5, 10). Our data show that 3 cases were transferred from surgery, gastroenterology, and other pediatric departments, and even one child was only accurately diagnosed during a second hospitalization. All children in our study had fever, 5 with accompanying abdominal pain and vomiting, only 2 with frequent urination and dysuria, and 1 child was hospitalized due to renal occupation.

In terms of laboratory data, our study found that the children's infection indicators were significantly elevated, such as peripheral blood white blood cell count, C-reactive protein, and procalcitonin, consistent with literature reports (4). Surprisingly, unlike acute pyelonephritis where urinary white blood cells are significantly higher than normal, we found that most children with renal abscesses had only a small amount of white blood cells in the urine, no pyuria, and a slight increase in urinary red blood cells. As literature reports, if the abscess does not break through the renal pelvis and calyces, and the pus does not enter the collecting system, obvious pyuria may not be present. Therefore, the presence and size of a renal abscess cannot be denied based on the degree of increase in urinary white blood cells and the presence or absence of pyuria (5, 7, 11).

Renal abscesses occur via different pathways, including ascending urinary tract and hematogenous infections, as well as inflammation spreading from adjacent organs. Ascending infections are often related to urogenital system malformations or recurrent urinary tract infections. The main pathogens are Gram-negative bacteria, predominantly Escherichia coli. Staphylococcal infections are the most common cause of hematogenous spread (12, 13). Lin Xiaoliang et al. reported a case of a child with a renal abscess where cultures were negative, and the pathogen was identified through metagenomic sequencing after ineffective treatment with third-generation cephalosporins. Urine and blood cultures in our group of children were all negative, which may be related to the abscesses being confined to the renal parenchyma and not breaking through the renal pelvis and calyces (7). Adult renal abscess patients often have underlying conditions such as diabetes, cirrhosis, female reproductive system infections, stress urinary incontinence, etc. (14, 15). It has been reported that children often have recurrent urinary tract infections, some of which are accompanied by urinary tract malformations (6) but children without underlying diseases can also develop renal abscesses (8). A Chinese study of 5 pediatric renal abscess cases found none with urogenital malformations, and 2 without recurrent urinary tract infections (16). Our group of children had no history of recurrent urinary tract infections, normal prenatal urinary system checks, and no history of urinary tract malformations or reflux. After infection control, cystourethrography was performed on 3 children, with one child found to have bilateral grade 3 reflux.

Due to the atypical clinical symptoms and lack of ultrasound, CT, and other imaging studies, it is difficult to make a definitive diagnosis based solely on clinical presentation and physical examination (13). Ultrasound, being simple to operate, radiation-free, non-invasive, affordable, and widely accepted by families, is often the first choice for initial examination. All 12 of our cases underwent ultrasound, but only 3 children showed abnormalities, suggesting that abscesses are hard to detect before abscess cavity formation and when the abscess is small, leading to missed diagnoses. After hospitalization, all children in our group completed CT or MRU scans. Due to the radiation of CT, children initially admitted to our department preferred magnetic resonance imaging, which showed good imaging results. Whether MRU will be preferred in the future requires further large-sample validation. Additionally, due to its simplicity, ultrasound can be used for clinical follow-up and assessment during the treatment of renal abscesses.

Based on acute pyelonephritis and previous literature reports of renal abscesses predominantly involving Gram-negative bacteria (2, 3, 5), treatment for our group of children upon admission initially involved third-generation cephalosporins. Since the children's blood and urine cultures were all negative, those whose temperature still fluctuated after 3–5 days of treatment were additionally treated with linezolid, achieving good results, with no similar reports in previous literature. Foreign literature reports the use of aminoglycoside antibiotics, which are almost never used in Chinese children (4, 12). There is still controversy regarding the treatment approach of conservative treatment vs. surgical intervention. Previous studies have shown that abscesses smaller than 3 cm can be treated with conservative anti-infection therapy, while those larger than 3 cm can be treated with percutaneous abscess puncture (2–4). A Chinese study showed significant therapeutic effects with conservative treatment for abscesses up to 4.7 cm in diameter (16). For cases 5 and 7, we initially considered abscess drainage. However, after adjusting antibiotics, the body temperature returned to normal, renal area tenderness was negative, and follow-up ultrasound showed the abscess had reduced in size. Therefore, abscess drainage was not performed, and conservative antibiotic treatment continued with good results, consistent with this study in our country.A Russian study retrospectively found that abscesses larger than 3 cm were treated with percutaneous abscess puncture and drainage (17). All 12 children in our group were treated conservatively, with a hospital stay of 10 days to 3 weeks and were discharged smoothly. 11 children were cured after 1–3 months of follow-up, the child with grade 3 reflux underwent surgical treatment for reflux, and 1 child showed a small abscess on ultrasound but had no clinical symptoms and normal urine routine.

This study highlights the atypical presentation and diagnostic challenges of pediatric renal abscesses. Fever, accompanied by non-specific abdominal or renal symptoms, should raise suspicion. The role of imaging, especially CT or MRU, is crucial in early diagnosis. Our findings suggest that conservative treatment with antibiotics is effective for small abscesses, while larger abscesses may require additional interventions.

## Conclusion

In summary, pediatric renal abscesses, though rare, should be considered in children presenting with fever and abdominal or renal symptoms. Early imaging and appropriate antibiotic treatment can lead to favorable outcomes, reducing the risk of complications.

This paper has some limitations: it is a single-center study with a small number of cases. Cultures were negative, and further metagenomic testing was not performed. Only some children underwent cystourethrography.
